# Metabolomic Analysis of The Chemical Diversity of South Africa Leaf Litter Fungal Species Using an Epigenetic Culture-Based Approach

**DOI:** 10.3390/molecules26144262

**Published:** 2021-07-14

**Authors:** Rachel Serrano, Víctor González-Menéndez, Germán Martínez, Clara Toro, Jesús Martín, Olga Genilloud, José R. Tormo

**Affiliations:** Fundación MEDINA, Av. Conocimiento 34, Health Sciences Technology Park, 18016 Granada, Spain; rachel.serrano@medinaandalucia.es (R.S.); victor.gonzalez@medinaandalucia.es (V.G.-M.); german.martinez@medinaandalucia.es (G.M.); clara.toro@medinaandalucia.es (C.T.); jesus.martin@medinaandalucia.es (J.M.); olga.genilloud@medinaandalucia.es (O.G.)

**Keywords:** fungal fermentations, epigenetic modifier, metabolomic, chemical diversity

## Abstract

Microbial natural products are an invaluable resource for the biotechnological industry. Genome mining studies have highlighted the huge biosynthetic potential of fungi, which is underexploited by standard fermentation conditions. Epigenetic effectors and/or cultivation-based approaches have successfully been applied to activate cryptic biosynthetic pathways in order to produce the chemical diversity suggested in available fungal genomes. The addition of Suberoylanilide Hydroxamic Acid to fermentation processes was evaluated to assess its effect on the metabolomic diversity of a taxonomically diverse fungal population. Here, metabolomic methodologies were implemented to identify changes in secondary metabolite profiles to determine the best fermentation conditions. The results confirmed previously described effects of the epigenetic modifier on the metabolism of a population of 232 wide diverse South Africa fungal strains cultured in different fermentation media where the induction of differential metabolites was observed. Furthermore, one solid-state fermentation (BRFT medium), two classic successful liquid fermentation media (LSFM and YES) and two new liquid media formulations (MCKX and SMK-II) were compared to identify the most productive conditions for the different populations of taxonomic subgroups.

## 1. Introduction

Microorganisms are known to be a rich source of new bioactive molecules with a wide range of biotechnological applications in the pharmaceutical, agrochemical and cosmetic industries. The rediscovery of already known structures, the usual low production yields and the difficulty in activating cryptic biosynthetic gene clusters (BGCs) under laboratory conditions are some of the limitations to exploit their huge chemical production capability [1,2,3]. Recent advances in laboratory automation and detection technologies, such as chromatography coupled to mass spectrometry, have contributed to the rapid and efficient generation of large libraries of natural products [2,3,4]. Moreover, different approaches through the variations in nutritional and culturing factors have been extensively applied in order to exploit the biosynthetic potential revealed from genome-based studies [5,6,7].

Fungi stand out for their ability to produce a high diversity of metabolites with biological activities as antibacterial, antifungal or cytotoxic agents [8,9,10,11,12]. Orders *Chaetothyriales*, *Dothideales*, *Eurotiales*, *Helotiales*, *Hypocreales* and *Pleosporales* are well known to produce a broad range of high chemically diverse active molecules [12]. In addition, fungi play an important role in organic matter decomposition and especially in leaf litter with complex processes of nutrient recycling [13,14]. Leaf litter is a well-studied environment, and many reports have revealed its great fungal diversity, dominated by *Ascomycota* species. The leaf litter fungal community also harbors a high proportion of rare species associated with the decomposition of wide diverse plants, invertebrates and animal excrements [14,15,16,17,18]. Among leaf litter studies, South Africa is a high biodiversity hotspot with multiple works focused on describing its fungal populations associated with leaf litter with many unique genera and species described: *Bactrodesmium* sp., *Brachydesmiella* sp., *Circinotrichum* sp., *Coniosporium* sp., *Periconiella* sp., *Sporidesmium* sp., *Parasarcopodium ceratocaryi* and *Rhexodenticula elegiae* [15,16,17,18].

Historically, variations in cultivation conditions have successfully been applied for exploiting the chemical diversity of fungal metabolism and increasing the chances of finding new bioactive molecules during fermentation processes [1,7,9,12]. The term “OSMAC” (One Strain Many Compounds), referred to modifications of media composition, aeration, culture format and temperature, is often applied on microbial fermentations to induce the production of additional compounds in response to these variables [1,9,12]. Besides the nutrient composition of fermentation media, the use of solid supports to promote cell differentiation and the addition of metabolic inductors are some approaches commonly used to enhance the probability for inducing changes in chemical profiles [4,8].

Transcription of BGCs is often controlled by epigenetic regulation such as histone deacetylation and DNA methylation. Small molecule epigenetic modifiers can modulate the microbial metabolism through the activation of cryptic biosynthetic pathways. Several reports have revealed the ability of epigenetic modifiers to alter the expression of silent or under-expressed BGCs, resulting in the modulation of the metabolomic profile and enhancement of the production of metabolites that were not synthetized under normal growth conditions [1,5,6,7,11,12,19,20,21,22,23,24,25,26,27]: Treatment of *Alternaria alternata* and *Penicillium expansum* with the histone deacetylase inhibitor trichostatin A resulted in a statistically significant increase in numerous unidentified metabolites for both species [21]. The addition of 5-azacytidine to cultures of *Cladosporium cladosporioides* stimulated the production of several oxylipins, and, when the culture was supplemented with suberoylanilide hydroxamic acid, it led to the isolation of two new perylenequinones (cladochromes F and G), four known cladochromes A, B, D, E and calphostin B [5]. Treatment of *Penicillium brevicompactum* with nicotinamide in static liquid fermentation induced the production of nine new compounds p-anisic acid, p-anisic acid methyl ester, benzyl anisate, syringic acid, sinapic acid, acetosyringone, phenyl acetic acid, gentisaldehyde and phydroxy benzaldehyde [23].

Suberoylanilide Hydroxamic Acid (SAHA) is one of the most frequently used inhibitors for inducing new bioactive metabolites in numerous epigenetic studies. The following metabolites were only produced when the strains were cultured with SAHA: Nygerone A produced by *Aspergillus niger* in semi-solid culture with vermiculite [24]; the anti-infective cytosporones by the marine fungus *Leucostoma persoonia* [25]; (10′S)-verruculide B, vermistatin and dihydrovermistatin induced in the endophytic fungus *Phoma* sp. nov. LG0217 [26]; two new natural sclerotioramine derivatives, isochromophilone XIV and XV, and two known compounds, sclerotioramine and (+)-sclerotiorin induced in the insect-associated fungi *Penicillium mallochii* CCH01 [27].

On another note, advances in Liquid Chromatography Mass Spectrometry (LC-MS) technologies have exponentially increased metabolomic studies. LC-MS detection provides complex information in a fast and robust way, with a high sensitivity to detect a wide range of microbial metabolites, establishing it as a very useful technology for improving early dereplication in drug discovery processes [2,4,28,29]. MASS Studio 2.3 is a software tool created by our group that allows the evaluation of the chemical diversity of microbial natural product libraries using large LC-MS datasets. This methodology simplifies the complexity of LC-MS data when comparing hundreds of samples by collapsing the time dimension and keeping the mass signal information [28,29]. MASS Studio allows multiple applications on metabolomics including, among others, the categorization of different fermentation conditions, the quantification of differentially induced metabolites, or the use of chemical metabolite information for the identification of species-specific chemotypes, a very useful tool for the prioritization of strains or metabolites for further studies [28,29].

The main goal of this study was to evaluate the effects of the addition of SAHA during the inoculum and fermentation stages, on a broad range of taxonomically diverse fungal strains isolated from leaf litter material collected in South Africa, where the influence of different fermentation conditions on the fungal metabolite production was characterized by the implementation of metabolomic analyses.

## 2. Results and Discussion

### 2.1. Fungal Fermentations

A taxonomically diverse group of fungal strains isolated from leaf litter samples of 17 local plants collected in South Africa was selected from the Fundación MEDINA Fungal Collection. A total of 232 fungal strains were identified and classified based on their morphology and ribosomal DNA sequences. As a result, more than 11 different fungal classes were identified, with *Agaricomycetes, Dothideomycetes*, *Eurotiomycetes*, *Leotiomycetes* and *Sordariomycetes* being the most representative. As many as 33 taxonomic orders, 77 families and more than 145 genera were identified, highlighting the large taxonomic diversity represented by fungi associated to leaf litter [14,15,16,17,18] (Table S1). The class *Dothideomycetes* grouped the highest number of fungal strains (*n* = 119), with members of the order *Pleosporales* (*n* = 94) as the most represented and diverse group of strains. When analyzed at the family level, this order included more than 20 families where *Didymellaceae* (*n* = 19), *Phaeosphaeriaceae* (*n* = 16), *Cucurbitariaceae* (*n* = 7) and *Didymosphaeriaceae* (*n* = 7) were the most predominant. The class *Sordariomycetes* (*n* = 34) was the second more representative class of the fungal population, with *Hypocreales* (*n* = 12) and *Xylariales* (*n* = 8) being the major orders. The classes *Leotiomycetes* (*n* = 28) and *Eurotiomycetes* (*n* = 15) were mainly represented by the orders *Helotiales* (*n* = 19) and *Chaetothyriales* (*n* = 7), respectively. All of basidiomycete strains belonged to the class *Agaricomycetes* (*n* = 11) including five different orders but with a low number of fungal strains. Other minor classes could be identified with a smaller number of strains that did not allow statistical determination of metabolomic trends: *Pezizomycetes* (*n* = 3), *Cystobasidiomycetes* (*n* = 1), *Microbotryosmycetes* (*n* = 1), *Orbiliomycetes* (*n* = 1), *Umbelopsidomycetes* (*n* = 1) and *Wallemiomycetes* (*n* = 1). Finally, due to their uniqueness, 17 fungal strains could not be assigned to any class and were classified as *Incertae sedis*.

**Figure 1:**
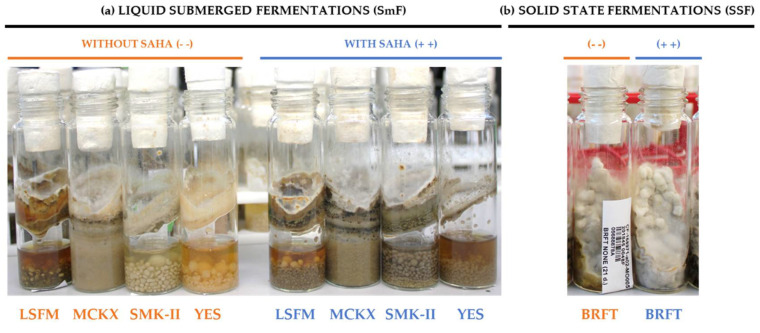
Example of *Parastagonospora* sp. CF-168971 showing different morphologies when grown in 10 fermentation conditions. (**a**) Fungal strain grown in liquid submerged fermentations (SmF) in four media LSFM, MCKX, SMK-II and YES without SAHA (- -) (four EPA vials on the left) and supplemented with SAHA (++) (four EPA vials on the right) after 14 days of incubation. (**b**) Fungal strain grown on solid-state fermentation (SSF) in BRFT medium without SAHA (- -) (EPA vial on the left) and with SAHA (++) (EPA vial on the right) after 21 days of incubation.

Each fungal strain was cultured in four different liquid fermentation media and one solid-state fermentation condition (SSF) to maximize the chances for inducing the production of secondary metabolites. The SSF BRFT medium was selected to include a rice-based medium, with brown rice as carbon source and yeast extract as a nitrogen source, with excellent reports on induction on chemical diversity. Regarding the liquid submerged fermentations (SmF), we selected two of the media due to their reported ability to enhance bioactive metabolite production (LSFM and YES) [9,10,11,12], and designed two new formulations for this work (MCKX and SMK-II). All of these fermentation conditions were evaluated with and without the epigenetic modifier SAHA (added during both the inoculum and fermentation stages) to evaluate the effects on the metabolite production. All of the studied fungal strains were well grown and many showed clear changes in color, texture and morphologies between the tested fermentation conditions, which could be indicative of changes in their secondary metabolite production (Figure 1a,b). After chemical extraction, a library of 2362 fungal extracts and blanks was obtained and compiled in 96-well plates, which were analyzed by LC-MS. The raw data files resulting from the different batches of analytical runs were processed with the MASS Studio tool, which generated a matrix of more than twenty-seven thousand different [rt-*m*/*z*] chemical components.

### 2.2. Influence of Media Composition on Fungal Fermentations

**Figure 2:**
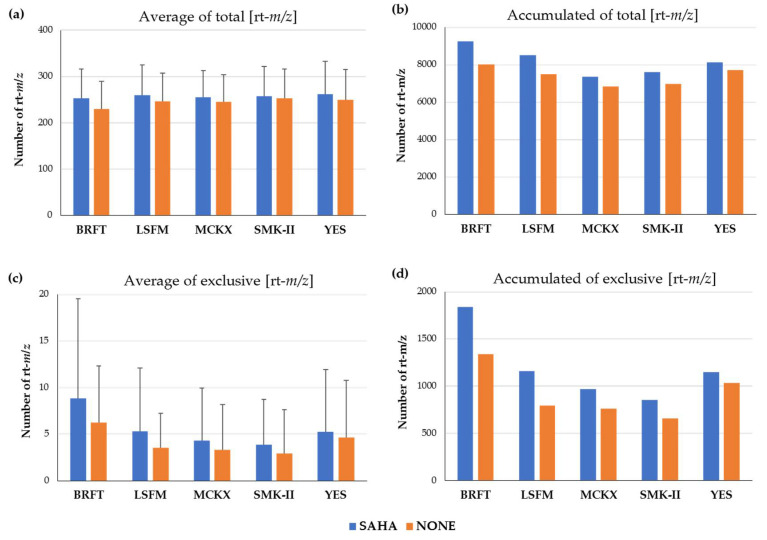
(**a**) Average of all [rt-*m*/*z*] components produced per strain in the five fermentation media with and without SAHA addition. (**b**) Accumulated differential components detected per fermentation medium. (**c**) Average of exclusive components produced per strain in the five fermentation media with and without SAHA addition. (**d**) Accumulated exclusive components detected per fermentation media. All values were calculated after subtraction of blank media components.

Secondary Metabolite (SM) profiles of the selected 232 fungal strains, grown in five fermentation media with and without SAHA, were compared in detail to identify differences between the growth conditions after subtraction of media components. Initially, no statistical differences in the average of the number of [rt-*m*/*z*] components detected per strain were observed for each of the five fermentation media (Figure 2a). All fermentation conditions seem to be contributing with a large number of metabolites ranging as average from 230 to 253 molecular components per strain. However, when the number of accumulated *m*/*z* produced by all fungal strains was compared, a slight increase was observed in BRFT fermentations. The SSF in BRFT medium showed higher total number of [rt-*m*/*z*] components for both SAHA (9253) and without SAHA (8022) conditions, always higher than the corresponding liquid conditions (Figure 2b). The number of exclusive different components produced with and without SAHA was also averaged per strain and medium (Figure 2c). Although fungal fermentations in BRFT resulted the best conditions, we could not statistically determine significant differences, probably due to the wide diversity of the selected fungal population. More interestingly, the accumulated number of exclusive components clearly highlighted BRFT medium as the most productive condition for the studied strains (Figure 2d). Specifically, fungal fermentations in BRFT with SAHA provided the highest number of [rt-*m*/*z*] components (1842), which were not detected in any of the other fermentation conditions. Previous studies have reported that SSF generally exhibits more complex metabolite profiles than the same strains when grown in liquid submerged conditions [4,8,30,31,32]. The use of SSF with fungi brings them closer to the conditions of their natural habitats, offering potential benefits for the microbial cultivation and metabolite production [30,31,32]. However, regarding whether the BRFT medium was a successful medium because of being a solid-state fermentation or because of its nutrient composition, and/or a synergy in both factors, it cannot be assured, since the different media were used in different culture systems. What can be confirmed is that BRFT with SAHA was the best medium for generating metabolomic changes on the wide taxonomically diverse fungal strains studied.

Regarding the four liquid fermentation media, two new designed media, MCKX and SMK-II, were prepared with specific media components in order to activate the huge metabolic capacity of the studied fungal population. On the one hand, MCKX medium has a rich base formulation which includes mannitol and yeast extract as carbon and nitrogen sources, ingredients used in other previously described production media MMK2 [10,11,12], but xylose was also added to present the same carbon source found in leaf litter environments, as it is the most abundant sugar in plant cell walls [33]. On the other hand, different carbon and nitrogen sources were selected for the fermentation medium SMK-II, including soluble starch, maltose and soybean flour, also reported to be components of successful formulations for bioactive metabolite production [10,11,12]. This fermentation medium was supplemented with L-proline, an amino acid that has been reported to be crucial in the production of antifungal compounds such as pneumocandin by *Glarea lozoyensis* [34]. Furthermore, Murashige and Skoog, a widely used plant growth medium that has been a common ingredient in some formulations for fungal growth [10,11,12,35], was added to both new production media MCKX and SMK-II to stimulate the growth and development of fungal strains able to metabolize leaf litter components. Classic LSFM and YES media, previously reported to be successful formulations to promote chemical changes during fungal fermentations and to enhance the production of fungal SMs [9,10,11,12], resulted to be the most productive conditions, followed by the new MCKX and SMK-II (Figure 2d). Despite fungal fermentations in both new media providing a lower number of exclusive and total [rt-*m*/*z*] components, no significative differences were observed when compared to the other classic liquid formulations. Therefore, both new formulations resulted effective to induce the production of a considerable range of unique metabolites.

### 2.3. Chemical Evaluation of SAHA Addition during Fungal Fermentations

**Figure 3:**
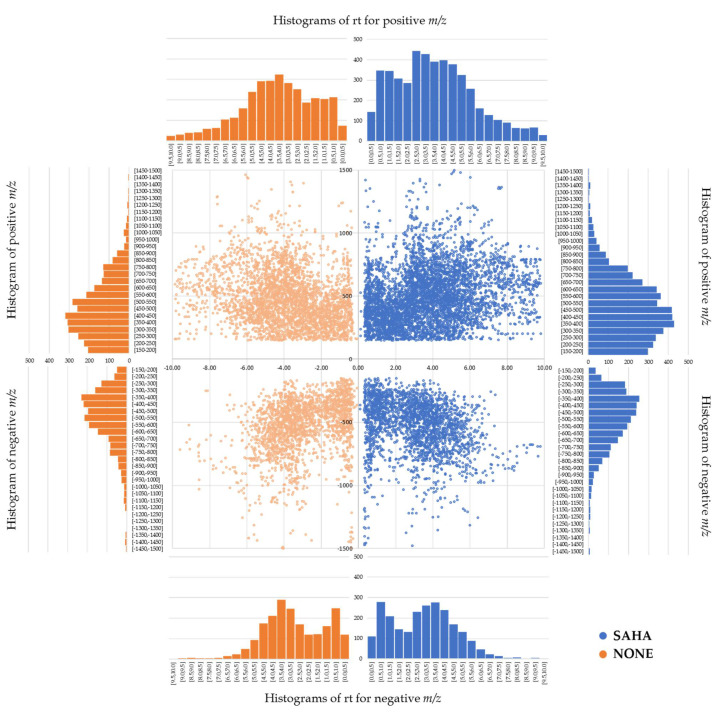
Distribution of all exclusive [rt-*m*/*z*] components for positive (**top**) and negative (**bottom**) ionization mode, detected in leaf litter fungal population fermented in the presence of SAHA (blue) and the absence of SAHA (orange).

Several studies have reported the use of epigenetic modifiers for inducing metabolomic changes during fungal fermentations through the activation of biosynthetic pathways that are silent under standard culture conditions [1,5,6,7,11,12,19,20,21,22,23,24,25,26,27]. Specifically, SAHA is the most used histone deacetylase inhibitor due to its capacity to modulate the fungal SM profiles and to induce the production of new metabolites in specific fungal strains [24,25,26,27]. However, the ability of SAHA to enhance the chemical diversity on a wide diverse fungal population set has never been analytically supported. In this work, the metabolomic effects of the SAHA addition during every fermentation stage (inoculum and production) for five different media were evaluated. In general, the addition of SAHA to the fermentation processes of the studied fungal population always improved the number of [rt-*m*/*z*] chemical components in every medium tested. Moreover, an increase in the exclusive components was also detected when SAHA was present during all fermentation processes (7132 [rt-*m*/*z*] components) in comparison to the same conditions without the addition of the epigenetic modifier (5267 [rt-*m*/*z*] components) (Figure 3).

Furthermore, the distribution of the exclusive [rt-*m*/*z*] components detected across the whole 232 fungal strains cultured with and without SAHA was compared after removing the common [rt-*m*/*z*] ones. In positive ionization mode of Mass Spectroscopy (MS), 4758 unique [rt-*m*/*z*] components were detected in fermentations with SAHA, whereas 3194 [rt-*m*/*z*] components were found in the same conditions without the epigenetic modifier. The total number of exclusive [rt-*m*/*z*] components detected in negative ionization mode, generally lower than positive, resulted to be 2374 with SAHA and 2073 without SAHA. The statistical mode of *m*/*z* values were similar in presence of SAHA for both detection modes (Table 1). However, the statistical mode was notably higher for positive ionization in conditions without SAHA. Regarding the retention times (rt), histograms show a bimodal distribution of data, where statistical mode intervals were similar between the conditions. Despite the discrepancies between positive and negative MS detection modes, generally related to the typical variances in the ionization of SMs [4], the addition of SAHA clearly improved the total number of exclusive [rt-*m*/*z*] components.

**Figure 4:**
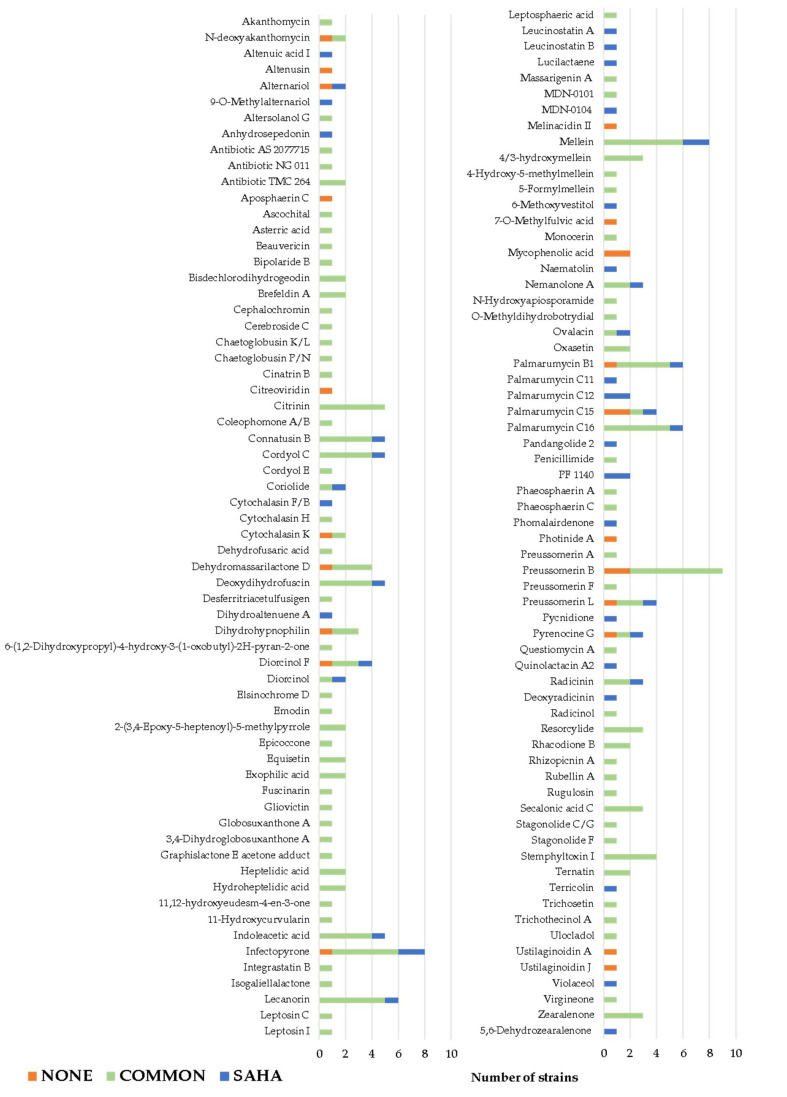
Distribution of known metabolites dereplicated for the leaf litter fungal population, only produced in fermentations supplemented with SAHA (blue), without SAHA (orange) and common to both conditions (green).

**Figure 5:**
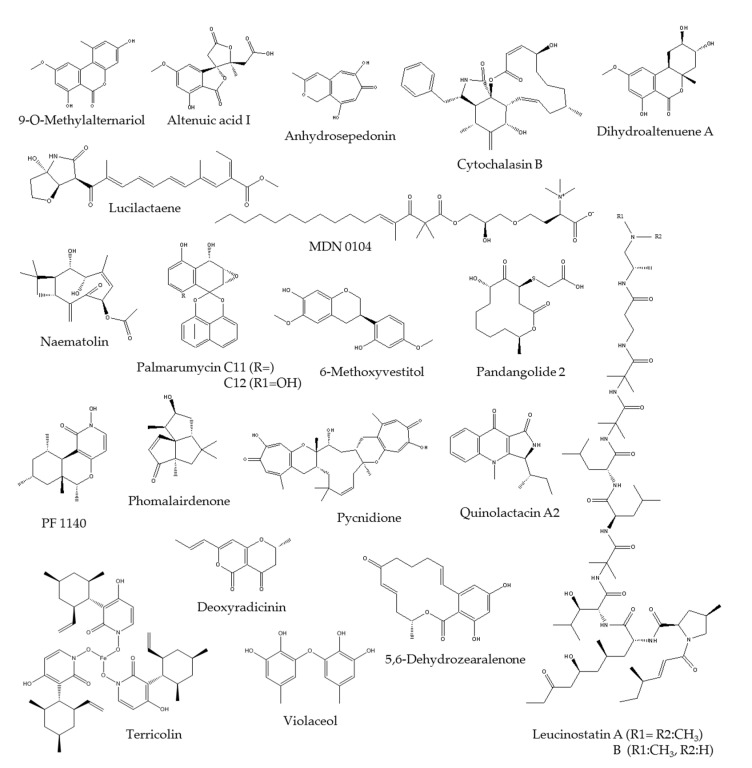
Chemical structures of dereplicated known metabolites induced by the addition of SAHA to fermentations of leaf litter fungal strain population.

Dereplication analyses by LC-MS and HR-MS were applied against MEDINA’s internal databases of known natural products and commercial database Dictionary of Natural Products (DNP), in order to evaluate further the effects of SAHA on the fungal fermentations [11,12,36]. As a result, 132 known metabolites (from 118 strains) were dereplicated in fungal fermentations with SAHA, without SAHA and common to both conditions (Figure 4, Table S1). Altenusin, Aposphaerin C, Citreoviridin, Melinacidin II, Mycophenolic acid, O-7-Methylfulvin acid, Photinide A as well as Ustilaginoidin A and J were only produced in fermentation conditions without SAHA. This is while 22 known metabolites with a high chemical diversity were only induced by the addition of SAHA to fungal fermentations: 9-O-Methylalternariol, Altenuic acid I, Anhydrosepedonin, Cytochalasin F or B, Dihydroaltenuene A, Leucinostatin A and B, Lucilactaene, MDN 0104, Naematolin, Palmarumycin C11 and C12, 6-Methoxyvestitol, Pandangolide 2, PF 1140, Phomalairdenone, Pycnidione, Quinolactacin A2, Deoxyradicinin, Violaceol, Terricolin and 5,6-Dehydrozearalenone (Figure 5). The remaining dereplicated molecules were produced constitutively by specific fungal strains cultured in conditions with and without SAHA.

**Figure 6:**
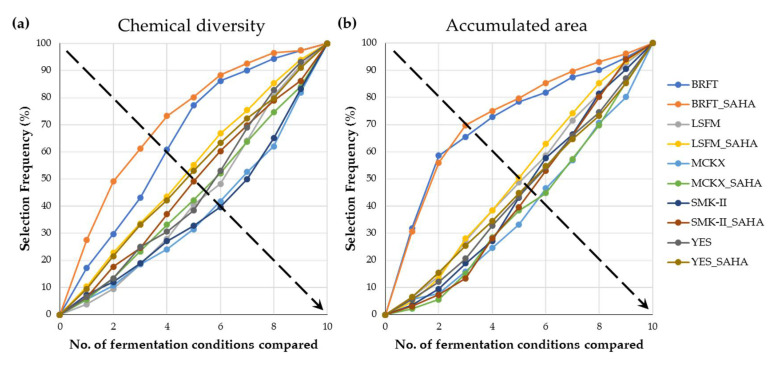
Ranking of all fermentation conditions studied in the total fungal population (*n* = 232); (**a**) based on the exclusive chemical diversity generated (presence or absence of [rt-*m*/*z*] components); (**b**) based on the amount of metabolites (as accumulated area of [rt-*m*/*z*] components).

In addition, MASS Studio 2.3 was implemented to characterize the chemical diversity and the amounts of metabolites produced by the addition of SAHA across all fungal fermentations. The software ranked and prioritized the different fermentation conditions from the best to the worst in inducing metabolomic changes [37,38,39]. The ranking processes consist of selecting the condition that generated the highest number of [rt-*m*/*z*] components as indicative of the chemical diversity achieved (Figure 6a), or the largest corresponding accumulated areas as indicative of the amount of produced SM (Figure 6b). Subsequently, the determination of the remaining condition that generates the highest number of components not previously contemplated or their accumulated areas are performed until all fermentation conditions have been ranked from highest to lowest diversity/quantity productions [9,10,37,38,39]. According to this methodology, divergence from the 1:1 diagonal lineal correlation indicates its difference from the average. Following this criterion, the fermentation conditions were ranked based on the generation of chemical diversity in BRFT+SAHA > BRFT > LSFM+SAHA > YES+SAHA > SMK-II+SAHA > MCKX+SAHA > YES > LSFM > SMK-II > MCKX (see from top left to bottom right following the diagonal arrow, Figure 6a). Regarding the accumulated area (production quantity), the liquid submerged fermentations are grouped close to the diagonal (Figure 6b); this indicates that the SmF conditions provided lower diversity and quantity of metabolites compared with the SSF for equivalent extracted biomasses. Both graphics show the BRFT solid medium in the upper part of the diagonal line, highlighting it as the most productive condition among the set. Furthermore, the addition of SAHA clearly influenced the ranking positions, so every fermentation medium with SAHA was selected in the ranking prior to its corresponding medium without the epigenetic modifier. These results, with such a large fungal population, have confirmed that the addition of SAHA during fermentation processes can activate the metabolomic capacity of a wide taxonomically diverse fungal population, in line with the reported effects of SAHA for individual fungal species [1,5,6,7,11,12,19,20,21,22,23,24,25,26,27]. Remarkably, none of the tested fermentation conditions was discarded by the software, confirming that all of them were effective for inducing changes in the metabolomic profiles of leaf litter fungal strains.

### 2.4. Effect of SAHA Addition to Different Taxonomic Orders

**Figure 7:**
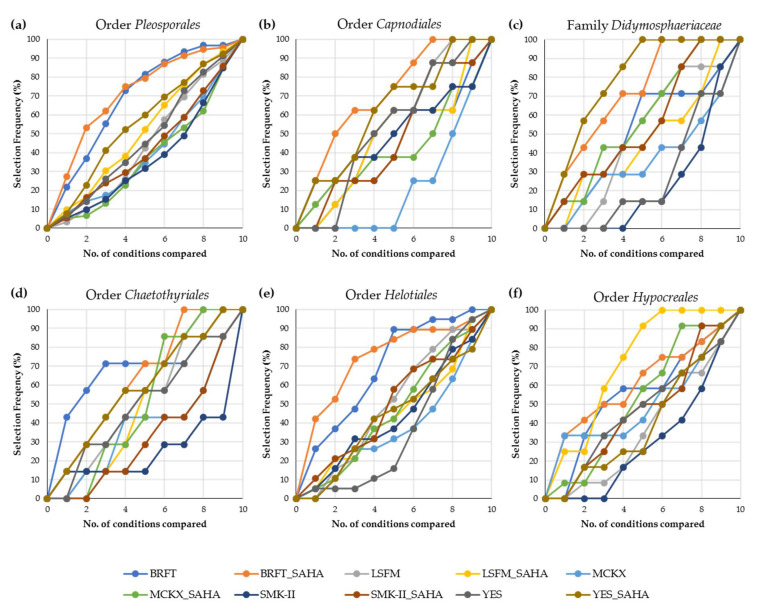
Rankings of the fermentation conditions based on the exclusive chemical diversity generated in different taxonomic groups: (**a**) order *Pleosporales* (*n* = 94), (**b**) order *Capnodiales* (*n* = 8), (**c**) family *Didymosphaeriaceae* (*n* = 7), (**d**) order *Chaetothyriales* (*n* = 7), (**e**) order *Helotiales* (*n* = 19) and (**f**) order *Hypocreales* (*n* = 12).

Due to the high taxonomic diversity of the studied fungal strains, we also focused on a detailed analysis of the potentially different effects of SAHA on taxonomic subgroups. For this purpose, we selected the most representative taxonomic orders within the major classes. In general, the solid-state fermentations in BRFT medium (with and/or without SAHA) were also able to provide the highest chemical diversity, being the first choice for the orders *Pleosporales*, *Capnodiales*, *Xylariales*, *Helotiales* and *Chaetothyriales* (Figure 7). In agreement with the general population, the ranking for each taxonomic group again showed that fungal fermentations with SAHA were ranked higher on presenting chemical diversity, compared with their corresponding conditions without the epigenetic modifier; however, some specific taxonomic groups showed some interesting differences.

Within the class *Dothideomycetes*, fermentations of the orders *Pleosporales* and *Capnodiales* in YES with SAHA stand out as the most productive SmF condition (Figure 7a,b), in line with the reported ability of this medium to produce a wide range of fungal metabolites [9,10]. Given that the order *Pleosporales* showed similar behavior to the general population (Figure 6a and Figure 7a) and was the most abundant of the studied fungal group (94/232), we decided to study in more detail the four major families within this order. Among them, the liquid medium YES supplemented with SAHA was determined to be a better condition than BRFT, only for the family *Didymosphaeriaceae*, followed by this SSF condition (Figure 7c).

In the case of the order *Chaetothyriales* (class *Eurotiomycetes*), fermentations in YES with SAHA were able to produce a high SM diversity similar to the one obtained by the SSF in BRFT with SAHA (Figure 7d), confirming the previously described condition for this medium. Only the order *Helotiales* (class *Leotiomycetes*) produced a high number of chemical components in the new formulation SMK-II supplemented with SAHA (Figure 7e). This behavior of *Helotiales* strains could be related to the reported ability of this fungal group to grow on maltose, one of the carbon sources used in the formulation of this medium [40].

By contrast, rankings of the class *Sordariomycetes* showed LSFM medium with SAHA as the best SmF condition for inducing chemical diversity in the two studied orders (*Xylariales* and *Hypocreales*), contrary to its corresponding medium without SAHA, which occupied the last positions. Interestingly, fermentations of *Hypocreales* strains in LSFM with SAHA were highlighted as the first selected condition for producing more chemical components than the SSF (Figure 7f). This is the unique taxonomic order in this study where an SmF supplemented with SAHA produced higher chemical diversity than the SSF, followed closely by the BRFT conditions (with and without SAHA).

**Figure 8:**
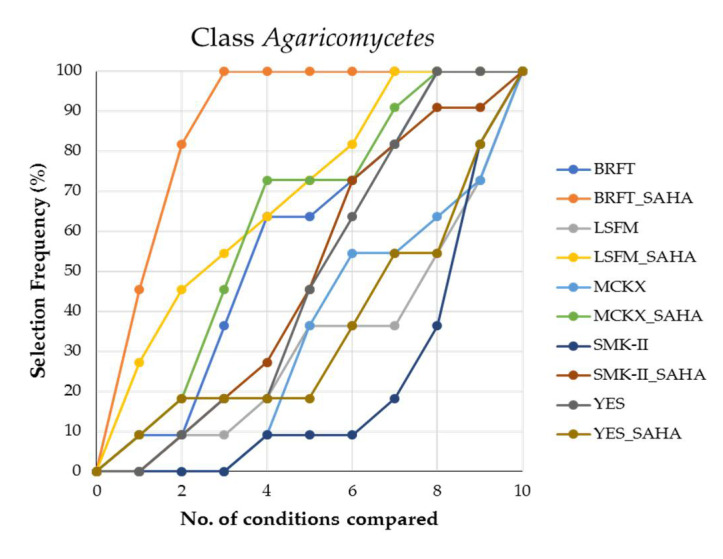
Ranking of the different conditions based on the exclusive chemical diversity generated from fermentations of the class Agaricomycetes (*n* = 11).

On another note, the class Agaricomycetes is one of the most important groups of leaf litter decomposers and includes quite different fungal strains with high metabolic potential [13]. Here, we studied a group of 11 basidiomycetes where the addition of SAHA to the fermentation media ensured a high improvement on the chemical diversity in BRFT, LSFM and MCKX media. Although the best fermentation condition was again BRFT with SAHA, the same medium without SAHA was unusually in the fourth place of the ranking (Figure 8). Moreover, fermentations of Agaricomycetes strains in MCKX with the epigenetic modifier significantly increased the chemical diversity, in contrast to the same medium without SAHA. The capacity of most of the basidiomycetes to degrade complex litter material could explain their ability to metabolize the xylose present in the formulation of the newly designed MCKX medium, producing a wide variety of metabolites [13].

### 2.5. Chemometrical Analysis of Chemical Profiles 

**Figure 9:**
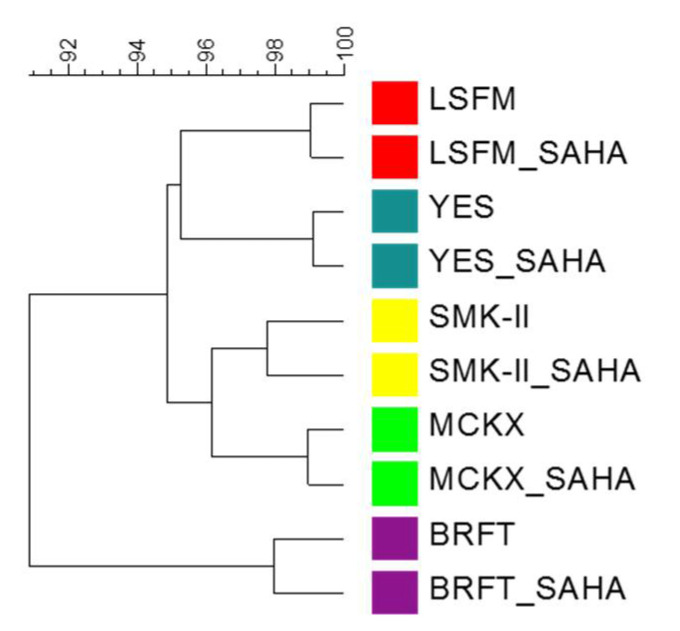
Dice-UPGMA dendrogram of similarity relationship between the metabolomic profiles of the fungal population (*n* = 232) cultured in five fermentation media with and without SAHA.

Cluster analysis using Dice similarity coefficient (presence or absence), with simple matching by UPGMA, was performed in order to represent the general similarity and relationships between SM profiles for the different fermentation conditions after removing media components. According to this statistical analysis, the dendrogram grouped the metabolomic profiles of the different fermentation media in pairs: with and without SAHA (Figure 9). In general, chemical profiles from the same fermentation medium with and without SAHA showed higher similarity percentages. Fungal fermentations in BRFT medium were highlighted again as the ones with the most different metabolomic profiles, compared with the remaining liquid conditions LSFM, MCKX, SMK-II and YES. The same statistical relationship was observed when the Pearson correlation coefficient, based on the area of [rt-*m*/*z*], was applied to the population (data not shown).

After the initial untargeted metabolomic studies by low resolution mass spectrometry data, subsequent detailed characterization of specific ions of interest (targeted metabolomics) by high resolution mass spectroscopy (HR-MS) was performed. HR-MS analyses allowed us to compare the chemical profiles generated from the addition of SAHA during fungal fermentations and to identify some of the enhancement effects in the metabolite production for specific strains in certain conditions. HR-MS provided more complex information and allowed to identify effects that included: (i) conditions where the addition of SAHA did not affect the metabolomic profile; (ii) conditions with clear improvement in the production titer of given compounds in the presence of SAHA; and/or (iii) compounds that only were produced in presence of SAHA.

**Figure 10:**
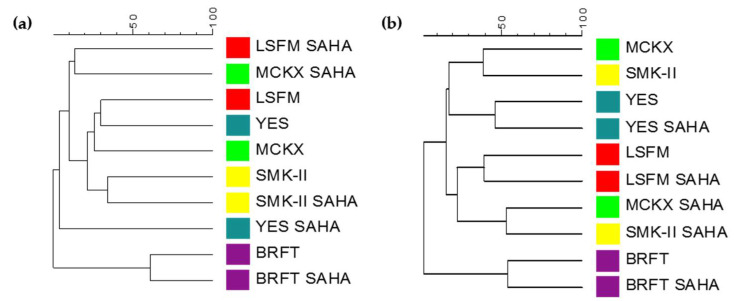
Dice-UPGMA dendrograms of metabolite profiles detected for the fungal strains; (**a**) *Corticium* sp. CF-166036 and (**b**) *Microxyphium* sp. CF-164412 grown in five fermentation media with and without SAHA.

**Figure 11:**
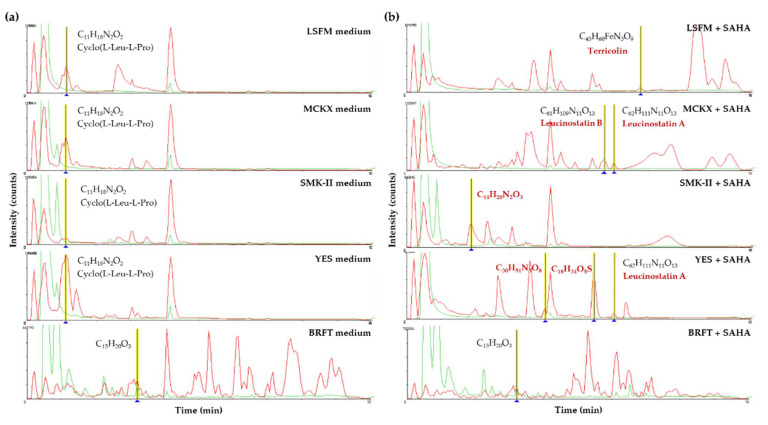
Comparative analysis of HR-MS profiles produced by fermentation of the strain *Corticium* sp. CF-166036 in five different media (**a**) without SAHA addition and (**b**) supplemented with SAHA. HPLC/UV at 210 nm profiles in green and HPLC/HR-MS profiles in red.

An example of metabolomic changes by the addition of SAHA is the strain *Corticium* sp. CF-166036, which showed some differences in the LC-MS profiles of the fermentation conditions when they were compared using the Dice similarity coefficient. Fermentations of this strain in BRFT and SMK-II media presented clear similarity between both conditions with and without SAHA. However, LSFM, MCKX and YES media showed metabolomic variations between conditions with and without SAHA, grouping them in different clusters of the dendrogram (Figure 10a). Moreover, interesting chemical changes were found after HR-MS profiles’ comparison (Figure 11). In general, fermentations of the strain *Corticium* sp. CF-166036 in the BRFT medium provided more complex HR-MS profiles in comparison to the other SmF conditions, but no significant differences were found between conditions with and without SAHA. Nevertheless, fermentations in the four liquid conditions LSFM, MCKX, SMK-II and YES with SAHA induced important changes in metabolite production. Several known metabolites that were induced by the addition of SAHA could be identified by comparing results with our internal database and the commercial Dictionary of Natural Products (DNP) [11,12,36]. This strain produced, in the presence of SAHA, Terricolin (*m*/*z* 842.367; C_45_H_60_FeN_3_O_9_) in LSFM, Leucinostatin B (*m*/*z* 1203.8174; C_61_H_109_N_11_O_13_) in MCKX and Leucinostatin A (*m*/*z* 1,217.8363; C_62_H_111_N_11_O_13_) in MCKX and YES media. In addition, three differential *m*/*z* detected could not be identified: *m*/*z* 264.1474 (C_14_H_20_N_2_O_3_) induced in MCKX with SAHA, as well as *m*/*z* 581.3664 (C_30_H_51_N_3_O_8_) and *m*/*z* 362.2119 (C_18_H_34_N_3_O_5_S), both induced in YES with SAHA.

**Figure 12:**
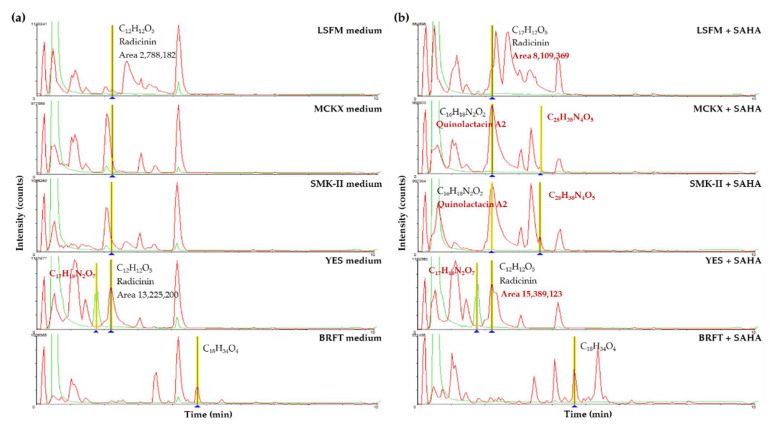
Comparative analysis of HR-MS profiles produced by fermentation of the strain *Microxyphium* sp. CF-164412 in five different media (**a**) without SAHA addition and (**b**) supplemented with SAHA. HPLC/UV at 210 nm profiles in green and HPLC/HR-MS profiles in red.

Another interesting example of metabolite induction during fermentation in the presence of SAHA is the strain *Microxyphium* sp. CF-164412. LC-MS profiles generated from fermentations in YES, LSFM and BRFT media showed similarities between their conditions with and without SAHA, grouping them in the same clusters (Figure 10b). By contrast, fermentations of this strain in the two new formulations supplemented with SAHA (MCKX and SMK-II media) induced important changes in their chemical profiles, placing both in different clusters distant from their corresponding conditions without SAHA. After comparing HR-MS profiles of this strain, several known and new metabolites were identified in the different fermentation conditions (Figure 12). The described Quinolactacin A2 (*m*/*z* 270.1362; C_16_H_18_N_2_O_2_) was detected only in MCKX and SMK-II media supplemented with SAHA. Moreover, two new metabolites without matches in our internal database or the DNP were identified; *m*/*z* 510.2801 (C_28_H_38_N_4_O_5_) was induced by the addition of SAHA to MCKX and SMK-II media and *m*/*z* 362.1076 (C_17_H_18_N_2_O_7_) was produced in YES medium with and without the addition of SAHA. Furthermore, a clear improvement in the amounts of Radicinin (*m*/*z* 236.0702; C_12_H_12_O_5_) was observed when this strain was grown in LSFM and YES media in the presence of SAHA.

Both examples demonstrated that the addition of SAHA during fermentations can enhance the production of specific metabolites in individual fungal strains, as well as improving the general diversity and the quantity of metabolites of a wide range of fungal strains.

## 3. Materials and Methods

### 3.1. Fungal Strains 

A taxonomically diverse subset of 232 fungal strains isolated from leaf litter collected in South Africa was selected for this study. Fungal strains were deposited in the Fundación MEDINA Culture Collection, one of the world’s most diverse and productive collections of filamentous fungi. Frozen cryotubes containing fungal inoculum or mycelia discs were cultured in Petri dishes containing YM agar as previously described [10,11,12]. After 7–14 days of incubation, five mycelial discs were transferred and crushed in the bottom of tubes containing 12 mL of SMYA medium in order to obtain a homogeneous mycelia inoculum of each strain. Inoculum tubes were supplemented by the epigenetic modifier Suberoylanilide Hydroxamic Acid (SAHA, TCI H1388), a histone deacetylases inhibitor. SAHA was dissolved in DMSO and adjusted at the final concentration of 100 µM in inoculum tubes and was incubated on an orbital shaker during 7 days at 22 °C and 70% RH [11].

### 3.2. Fermentation Conditions

Fungal cultures were inoculated by the addition of 0.3 mL of inoculum into EPA vials containing the five production media with the different carbon and nitrogen sources and fermentation states (SmF and SSF). Three of the selected culture media had been commonly used in fungal fermentation: BRFT medium (SSF) [41], LSFM medium and YES medium (SmF) [10,11,12]. Two new formulations have been developed in this work: MCKX medium (mannitol VWR 25311.297 40 g/L, corn meal yellow SIGMA C6304 10 g/L, yeast extract DIFCO 212750 5 g/L, Murashige and Skoog salts SIGMA M5524 4.3 g/L, xylose SIGMA X1500 10 g/L and deionized water to 1 L) and SMK-II medium (soluble starch from potato PANREAC 121096 40 g/L, maltose FISHER BP 684-500 40 g/L, soybean flour SIGMA S9633 1 g/L, L-proline MERCK 1.07434.0500 3 g/L, Murashige and Skoog salts SIGMA M-5524 4.3 g/L and deionized water to 1 L). SAHA dissolved in DMSO was added to each EPA vial at the final concentration of 100 µM [11]. Fermentations were incubated at 22 °C and 70% RH for 14 days with the SmF (LSFM, MCKX, SMK-II and YES) media and for 21 days with the BRFT SSF medium. 

### 3.3. Chemical Extraction

After incubation time, fermentation broths from the liquid media were extracted by adding equal volume of acetone [10,11,12] or equivalent volume of methyl-ethyl-ketone (MEK) for solid fermentation, as previously described [30,41], using a Multiprobe II robotic liquid handler (PerkinElmer, Waltham, MA, USA) and shaking at 220 rpm for 1 h. Acetone, miscible with water, has been described as the solvent of choice for whole broth extractions of liquid submerged fermentations [10,11,12], whereas MEK, immiscible with water, extracts just the region of medium to low polarity compounds, but has been reported to be more successful on ensuring maximum extraction of SM for solid state fermentations [9]. The samples were centrifugated, and 12 mL of supernatant from each vial was transferred to glass tubes containing 0.6 mL of DMSO and mixed. The amount of 5 mL of water was added to the solid fermentation extracts and organic solvents, and parts of water were evaporated under a heated nitrogen stream to a final volume of 3 mL (80/20 water/DMSO solution), along with a final concentration of 2 × WBE [10]. Each fermentation batch included extracts from control media to discriminate their components.

### 3.4. LC-MS Profile Analysis and Database Matching of Known Metabolites

Fermentation extracts (4 µL) were analyzed by Liquid Chromatography Mass Spectrometry (LC-MS), with a 10 min gradient using a Zorbax SB-C8 column (3.5 µm; 2.1 × 30 mm), on an Agilent 1100 single Quadrupole MSD Low Resolution Mass Spectrometer (Santa Clara, CA, USA). Solvent A consisted of 10% acetronitrile and 90% water with 0.01% trifluoroacetic acid and of 1.3 mM ammonium formate, while solvent B was 90% acetronitrile and 10% water with 0.01% trifluoroacetic acid and 1.3 mM ammonium formate [10,11,12]. The gradient started at 10% of solvent B and went to 100% in 6 min, kept at 100% of solvent B for 2 min and returned to 10% for 2 min to initialize the system, maintained at 40 °C and with a flow rate of 300 µL/min [10,11,12]. Full diode array UV scans from 100 to 900 nm were collected in 4 nm steps at 0.25 sec/scan. Ionization of eluting solvent was obtained using the standard Agilent 1100 ESI source adjusted to a drying gas flow of 11 L/min at 325 °C and a nebulizer pressure of 40 psig. The capillary voltage was set to 3500 V. Mass spectra were collected as full scans from 150 to 1500 *m*/*z*, with one scan every 0.77 s, in both positive and negative modes. Methanol blanks were injected every 80 samples for monitoring each analytical batch.

The obtained chemical raw data consisted in two sets of [rt-*m*/*z*] data for each extract in negative and positive ionization modes [28,29]. The sample information regarding each fungal strain, the fermentation medium, additives, extraction procedures and the plate map position in the storage plates were recorded in an Oracle system database (Nautilus LIMS by Thermo) [10,28,29,37,38,39]. 

Database matching was performed using an in-house developed application where the DAD, retention time, positive and negative mass spectra of the samples were compared to the UV-LC-MS data of known metabolites, stored in a proprietary database where metabolite standard data were obtained using the exact same LC-MS conditions as the samples under analysis [36].

### 3.5. MASS Studio 2.3 Processing

The LC-MS raw data files were managed in the MASS Studio 2.3 software. This tool allowed the comparison of the [rt-*m*/*z*] components detected in all injected samples and to correlate to their corresponding control media [28,29]. Characterization of the generated library of extracts allowed the ranking of the different tested fermentation conditions as previously described [37,38,39]. The prioritization process consisted in the sequential selection of the medium which generated the highest number of [rt-*m*/*z*] components or accumulated area of [rt-*m*/*z*], indicating the highest chemical diversity achieved and the amount of detectable material [10,37,38,39]. The metabolomic data matrixes were statistically analyzed with the commercial BioNumerics^®^ 6.6 software (Applied Maths^™^) to generate similarity matrixes according to Dice (presence/absence) and Pearson (area/intensity) coefficients and simple matching UPGMA. 

### 3.6. HR-MS Profile Analysis and Database Matching of Known Metabolites

Selected extracts (2 µL) were analyzed by HPLC/HR-MS on an Agilent (Santa Clara, CA, USA) 1200, using a Zorbax SB-C8 column (2.1 × 3.0 mm), maintained at 40 °C with a flow rate of 300 µL/min. Solvent A consisted of 10% acetonitrile and 90% water with 0.01% trifluoroacetic acid and 1.3 mM ammonium formate, while solvent B was 90% acetonitrile and 10% water with 0.01% trifluoroacetic acid and 1.3 mM ammonium formate. The gradient started at 10% of solvent B and went to 100% in 6 min, kept at 100% of B for 2 min and returned to 10% B for 2 min to initialize the system. Full diode array UV scans from 100 to 900 nm were collected in 4 nm steps at 0.25 sec/scan. 

Mass spectrometry acquisition was performed on a Bruker maXis HR-QTOF mass spectrometer (Bruker Daltonics GmbH, Bremen, Germany) coupled to the previously described LC system. Ionization of the eluting solvent was obtained using the standard maxis ESI source adjusted to a drying gas flow of 11 L/min at 200 °C and a nebulizer pressure of 40 psig. The capillary voltage was set to 4000 V. Mass spectra were collected from 50 to 2000 *m*/*z* in positive mode. 

Chemical profiles were compared to MEDINA’s internal database of known natural products by fingerprint matching of their retention time, ultraviolet signal and mass spectrometry data [11,12,36]. Compounds that were not identified were further analyzed by HR-MS for tentative identification by matching their predicted molecular formulae with the commercial Chapman & Hall Dictionary of Natural Products (DNP).

## 4. Conclusions

In summary, this study is the first systematic metabolomic analysis of the effects of SAHA on nutritional arrays of a large number of taxonomically diverse fungal strains cultured in solid and liquid state conditions. The results confirmed that changes in the medium composition influenced largely in their metabolomic profiles. Fermentation conditions were ranked in order to determine the best conditions to improve SM diversity production for each taxonomic group. This was the first chemometric confirmation that supports the solid-state fermentation BRFT as the most productive formulation that generated greater metabolomic changes, which is in line with the described benefits of its culturing for a significant enhancement of SM production. The results also supported the hypothesis that the addition of SAHA during fungal fermentations can determine substantial metabolomic changes of the chemical profiles for entire fungal populations as well as for different taxonomic classes, orders or families. Furthermore, studies on the evaluation of the potential activity of the generated library of extracts are currently in progress, besides the isolation and characterization of the most interesting and unknown metabolites induced by the addition of SAHA to specific fungal fermentations.

## Figures and Tables

**Table 1:** Statistical mode value intervals calculated for each histogram of positive and negative ionization detection modes in Mass Spectroscopy (MS), based on the population of *m*/*z* and retention time (rt) values.

	SAHA	NONE
**Exclusive *m*/*z* (MS positive mode)**	[350, 400]	[400, 450]
**Exclusive *m*/*z* (MS negative mode)**	[−350, −400]	[−350, −400]
**Exclusive rt (MS positive mode)**	[0.5, 1.0], [2.5, 3.0]	[0.5, 1.0], [3.5, 4.0]
**Exclusive rt (MS negative mode)**	[−0.5, −1.0], [−3.5, −4.0]	[−0.5, −1.0], [−3.5, −4.0]

## Data Availability

The data presented in this study are available in Supplementary Material.

## Supplementary Materials

The following are available online. Table S1: Taxonomical classification of the selected 232 fungal strains isolated from leaf litter of different local plants collected in South Africa across classes, orders, families and genus. Highlighted in bold are the subsets studied in detail.

## Sample Availability

Samples are available from the authors.
