# Supplementary material for: Metabolomic Analysis of The Chemical Diversity of South Africa Leaf Litter Fungal Species Using an Epigenetic Culture-Based Approach
Source: Molecules. 2021 Jul 14;26(14):4262. doi: 10.3390/molecules26144262 (PMC8305139; doi:10.3390/molecules26144262)
Supplement: Supplementary file 1 [file molecules-26-04262-s001.zip › molecules-1271159-supplementary.pdf]

Supplementary Materials

Metabolomic Analysis of the Chemical Diversity of Leaf Litter Fungal Species Using an Epigenetic Culture-Based Approach

Rachel Serrano, Víctor González-Menéndez, Germán Martínez, Clara Toro, Jesús Martín, Olga Genilloud and José R. Tormo*

Fundación MEDINA, Av. Conocimiento 34, Health Sciences Technology Park, 18016 Granada, Spain
*Correspondence: ruben.tormo@medinaandalucia.es; Tel.: +34 958 993 965

Table S1. Taxonomical classification of the selected 232 fungal strains isolated from leaf litter of different local plants collected in South Africa across classes, orders, families and genus. Highlighted in bold the subsets studied in detail.

Class	Tax Order	Family	Genus	Origin	Dereplicated known metabolites
Agaricomycetes	*Corticiales*	*Corticiaceae*	*Corticium* (n=2)	*Protea laurifolia* and *P. repens*	Connatusin B, Leucinostatin A and B, Terricolin
			Marchandiomyces	*Thamnochortus* sp.	
	Cantharellales	*Hydnaceae*	*Sistotrema*	*Protea repens*	
		Tulasnellaceae	*Tulasnella*	Culm restio	
		Incertae sedis	*Chantharealles*	*Olea europaea sbsp africana*	
	Polyporales	*Phanerochaetaceae*	*Phanerochaete*	*Rafnia cf. triflora* and *Tarchonanthus camphorates*	
		Polyporaceae	*Trametes*	Oak tree *Quercus* sp.	
	Agaricales	*Physalacriaceae*	*Cryptomarasmius*	*Protea repens*	Diorcinol
		Psathyrellaceae	*Coprinellus*	*Sideroxylon inerme*	
	Boletales	*Serpulaceae*	*Serpula*	*Brabejum stellatifolium*	
Dothideomycetes	***Pleosporales***	***Didymellaceae***	*Ascochyta* (n=5)	*Olea europaea sbsp africana*	Brefeldin A, Gliovictin, Indoleacetic acid, Leptosin I and C, Massarigenin A, Ovalacin, Palmarumycin C11 and C12
			Boeremia	*Chrysanthemoides monilifera*	
			Didymella (n=3)	*Elegia capensis* and culm restio	
			Neodidymelliopsis	*Protea repens*	
			Phoma (n=5)	*Protea repens* and culm restio	Cordyol C, Lecanorin, Phomalairdenone
		Phaeosphaeriaceae	*Banksiophoma*	Restio cf. *multiflorus*	Aposphaerin C
			Neosetophoma	Fynbos of restios	
			Neostagonospora	Fynbos of restios	Resorcylide
			Nodulosphaeria	*Olea europaea sbsp africana*	Dehydromassarilactone D, Integrastatin B, Nemanolone A, Palmarumycin B1
			Parastagonospora (n=3)	*Olea europaea sbsp africana*	Cordyol C, Dihydrohypnophilin, Stagonolide F and C/G
			Phaeosphaeria (n=4)	*Olea europaea sbsp africana*	Dihydrohypnophilin, Mellein, 2-(3,4-Epoxy-5-heptenoyl)-5-methylpyrrole, Phaeosphaerin A and C, 11,12-hydroxyeudesm-4-en-3-one
			Sclerostagonospora (n=2)	*Elegia capensis*	Resorcylide, Zearalenone, 2-(3,4-Epoxy-5-heptenoyl)-5-methylpyrrole)
			Unidentified Phaeosphaeriaceae (n=2)	*Olea europaea sbsp africana*	Infectopyrone, Penicillimide

Dothideomycetes	***Pleosporales***	***Phaeosphaeriaceae***	*Wojnowiciella*	*Carpobrotus edulis*	Bisdechlorodihydrogeodin, Pycnidione
		Cucurbitariaceae	*Neocucurbitaria* (n=2)	*Protea laurifolia*	Infectopyrone, 6-(1,2-Dihydroxypropyl)-4-hydroxy-3-(1-oxobutyl)-2H-pyran-2-one
			Pyrenochaeta (n=2)	Culm restio	Indoleacetic acid, Infectopyrone
			Pyrenochaetopsis (n=3)	*Chrysanthemoides monilifera* and culm restio	Preussomerin L
		Didymosphaeriaceae	*Didymosphaeria*	*Protea laurifolia*	Connatusin B, Diorcinol F, Deoxydihydrofuscin
			Paracamarosporium	*Protea laurifolia*	
			Paraconiothyrium (n=2)	*Protea laurifolia*	Emodin, Globosuxanthone A, Dihydroglobosuxanthone A
			Paraphaeosphaeria (n=2)	*Protea laurifolia*	Cordyol C
			Pseudocamarosporium	*Olea europaea sbsp africana*	Deoxydihydrofuscin
		Massarinaceae	*Helminthosporium*	Fynbos of restios	
			Lophiostoma (n=2)	Culm restio	Indoleacetic acid, Preussomerin A and B, Palmarumycin B1, C15 and C16
			Massarina	Restio cf. *multiflorus*	Dehydromassarilactone D, Infectopyrone, Pyronecine G
			Vaginatispora (n=2)	Culm restio	Equisetin, Oxasetin
		Teichosporaceae	*Teichospora* (n=3)	Culm restio	
			Teichosporaceae	Culm restio	
		Coniothyriaceae	*Coniothyrium* (n=3)	*Sideroxylon inerme* and culm restio	
		Pleosporaceae	*Alternaria* (n=3)	*Protea repens*	Alternariol, 9-O-Methylalternariol, Altenusin, Altenuic acid, Dihydroaltenuene A, Infectopyrone, 7-O-Methylfulvic acid, Pyrenocine G, Violaceol
		Sporormiaceae	*Forliomyces (n=3)*	*Elegia capensis* and culm restio	Mellein
		Amorosiaceae	*Alfoldia*	*Rafnia cf. triflora* and *Tarchonanthus camphorates*	
			Angustimassarina	Oak tree *Quercus* sp.	Rugulosin
		Anteagloniaceae	*Anteaglonium* (n=2)	Oak tree *Quercus* sp.	Ascochital
		Cryptocoryneaceae	*Cryptocoryneum* (n=2)	Culm restio	Palmarumycin B1, C15 and C16, Preussomerin B
		Microsphaeropsidaceae	*Microsphaeropsis* (n=2)	*Thamnochortus* sp.	
		Biatriosporaceae	*Nigrograna*	*Protea repens*	
		Delitschiaceae	*Delitschia*	*Sideroxylon inerme*	

Dothideomycetes	***Pleosporales***	*Hermatomycetaceae*	*Hermatomyces*	*Protea repens*	MDN-0104, Mycophenolic acid, Rubellin A
		Lentitheciaceae	*Keissleriella*	Fynbos of restios	
		Leptosphaeriaceae	*Leptosphaeria*	*Protea laurifolia*	Leptosphaeric acid
		Libertasomycetaceae	*Libertasomyces*	*Diosma subulata*	Akanthomycin
		Roussoellaceae	*Roussoella*	*Diosma subulata*	PF 1140
		Thyridariaceae	*Lophiostoma*	Culm restio	Bipolaride B, Oxasetin
		Torulaceae	*Dendryphiella*	Culm restio	
		Incertae sedis	*Parapyrenochaeta*	*Elegia capensis*	Infectopyrone
			Unidentified Pleosporales (n=8)	Culm restio	Dehydromassarilactone D, Dihydrohypnophilin, Diorcinol F, Indoleacetic acid, Lecanorin, Pyrenocine G, Radicinin, Deoxyradicinin, Radicinol
	Capnodiales	*Teratosphaeriaceae*	*Austroafricana* (n=3)	*Olea europaea sbsp africana* and *Protea repens*	
			Neocatenulostroma	Oak tree *Quercus* sp.	
			Neophaeothecoidea	*Protea laurifolia*	
			Parateratosphaeria	*Rafnia cf. triflora* and *Tarchonanthus camphorates*	
		Cladosporiaceae	*Cladosporium*	Culm restio	
			Verrucocladosporium	*Carpobrotus edulis*	
	Dothideales	*Dothioraceae*	*Dothiora* (n=3)	Oak tree *Quercus* sp.	Coriolide, Coleophomone A/B, Lecanorin, Ovalacin
			Kabatina	Culm restio	Palmarumycin C12
		Saccotheciaceae	*Dothichiza*	Oak tree *Quercus* sp.	Monocerin
	Botryosphaeriales	*Botryosphaeriaceae*	*Neofusicoccum*	*Protea repens*	O-Methyldihydrobotrydial
		Incertae sedis	*Camarosporium* (n=2)	*Diosma subulata* and *Elegia capensis*	Cordyol C and E, Deoxydihydrofuscin Lecanorin
	Venturiales	*Venturiaceae*	*Anungitea*	*Protea laurifolia*	Secalonic acid C
			Venturia	*Olea europaea sbsp africana*	
	Hysteriales	*Hysteriaceae*	*Hysterium*	*Thamnochortus* sp.	
	Superstratomycetales	*Superstratomycetaceae*	*Superstratomyces*	*Protea laurifolia*	
	Tubeufiales	*Incertae sedis*	*Neorhamphoria*	*Protea laurifolia*	
	Incertae sedis	*Pseudoperisporiaceae*	*Nematostoma*	*Protea repens*	
		Incertae sedis	*Pyrenochaeta*	Culm restio	Infectopyrone
			Scleroconidioma (n=2)	*Protea laurifolia* and *P. repens*	Antibiotic AS 2077715
Leotiomycetes	***Helotiales***	*Arachnopezizaceae*	*Arachnopeziza* (n=2)	*Olea europaea sbsp africana*	Altersolanol G, Citreoviridin, Citrinin, Cytochalasin F/B, Radicinin, Rhacodione B, Graphislactone E acetone adduct

Leotiomycetes	***Helotiales***	*Discinellaceae*	*Fontanospora*	*Brabejum stellatifolium*	Virgineone
		Hamatocanthoscyphaceae	*Chalara*	Oak tree *Quercus* sp.	
		Hamatocanthoscyphaceae	*Xenopolyscytalum*	*Olea europaea sbsp africana*	Rhacodione B
		Helotiaceae	*Hymenoscyphus*	*Olea europaea sbsp africana*	
			Lanzia	*Carpobrotus edulis*	
		Hyaloscyphaceae	*Meliniomyces*	Oak tree *Quercus* sp.	
		Loramycetaceae	*Acidomelania*	*Protea repens*	Citrinin
		Mollisiaceae	*Mollisia*	*Protea repens*	
		Sclerotiniaceae	*Stromatinia*	Fynbos of restios	
		Solenopeziaceae	*Tetracladium*	Culm restio	
		Incertae sedis	*Phialea*	*Protea laurifolia*	
			Unidentified Helotiales (n=6)	*Protea laurifolia, P. repens,* Oak tree *Quercus* sp., *Olea europaea* sbsp *africana*	Citrinin
	Rhytismatales	*Calloriaceae*	*Dactylaria* (n=2)	*Protea repens* and culm restio	PF 1140
		Rhytismataceae	*Lophodermium*	Fynbos of restios	
	Erysiphales	*Amorphothecaceae*	*Oidiodendron* (n=2)	Oak tree *Quercus* sp.	Asterric acid, Bisdechlorodihydrogeodin, 4/3-hydroxymellein, Dehydromassarilactone D, Deoxydihydrofuscin, Diorcinol F, Epicoccone, Fuscinarin, Trichothecinol A
	Incertae sedis	*Neocrinulaceae*	*Neocrinula*	*Protea repens*	
		Incertae sedis	*Coleophoma* (n=2)	*Protea repens* and *Olea europaea* sbsp *africana*	
		Calloriaceae	*Meliniomyces*	*Olea europaea* sbsp *africana*	Brefeldin A
Sordariomycetes	***Hypocreales***	*Nectriaceae*	*Cosmospora*	Oak tree *Quercus* sp.	Cephalochromin, Diorcinol F, Ustilaginoidin A and J
			Dactylonectria	*Sideroxylon inerme*	
			Fusarium (n=3)	Culm restio	Beauvericin, Dehydrofusaric acid, 5,6-Dehydrozearalenone, Equisetin, Lucilactaene, 6-Methoxyvestitol, Trichosetin
			Fusicladium	Culm restio	
			Volutella	*Sideroxylon inerme*	
		Bionectriaceae	*Bryocentria*	*Sideroxylon inerme*	
		Hypocreaceae	*Acrostalagmus*	*Protea repens*	Exophilic acid, Melinacidin II
		Stachybotryaceae	*Sirastachys*	*Rafnia* cf. *triflora* and *Tarchonanthus camphorates*	Mellein
		Incertae sedis	*Acremonium*	*Protea repens*	Deoxydihydrofuscin
			Sarocladium	*Protea laurifolia*	

Sordariomycetes	***Xylariales***	*Xylariaceae*	*Anthostomella* (n=2)	*Sideroxylon inerme*	Anhydrosepedonin, 5-Formylmellein, Heptelidic acid, Hydroheptelidic acid, Ternatin,
			Anthostomelloides	*Protea repens*	Cytochalasin K and H, Heptelidic acid, Hydroheptelidic acid, Ternatin
			Circinotrichum (n=2)	*Carpobrotus edulis* and *Sideroxylon inerme*	Antibiotic TMC 264, Cinatrin B, Connatusin B, Rhizopicnin A
		Diatrypaceae	*Monosporascus*	*Thamnochortus* sp.	4-Hydroxy-5-methylmellein
		Hypoxylaceae	*Daldinia*	*Sideroxylon inerme*	Indoleacetic acid
		Incertae sedis	*Pleurophoma*	Fynbos of restios	
	Amphisphaeriales	*Bartaliniaceae*	*Bartalinia* (n=2)	*Diosma subulata* and *Sideroxylon inerme*	Lecanorin, Mellein, 4/3-hydroxymellein, N-deoxyakanthomycin
		Amphisphaeriaceae	*Immersidiscosia*	*Olea europaea* sbsp *africana*	Antibiotic TMC 264, Secalonic acid C, Ulocladol
		Discosiaceae	*Discosia*	*Brabejum stellatifolium*	Naematolin, Photinide A
		Phlogicylindriaceae	*Phlogicylindrium*	*Olea europaea sbsp africana*	
		Sporocadaceae	*Sarcostroma*	*Protea repens*	Diorcinol, Nemanolone A
	Togniniales	*Togniniaceae*	*Phaeoacremonium*	*Rafnia cf. triflora* and *Tarchonanthus camphorates*	Desferritriacetulfusigen
			*Tognini*a	*Protea repens*	
	Myrmecridiales	*Incertae sedis*	*Myrmecridium*	Fynbos of restios	
	Sordariales	*Incertae sedis*	*Unidentified Sordariales*	*Olea europaea* sbsp *africana*	
	Incertae sedis	*Apiosporaceae*	*Arthrinium* (n=3)	*Thamnochortus* sp. and *Elegia capensis*	Alternariol, Brefeldin A, Cordyol C, Cytochalasin K, N-Hydroxyapiosporamide,
		Incertae sedis	*Pleurophoma*	Fynbos of restios	
Eurotiomycetes	***Chaetothyriales***	*Herpotrichiellaceae*	*Capronia*	*Diosma subulata*	Exophilic acid
			Cladophialophora	*Protea laurifolia*	Mellein
			Exophiala (n=3)	*Protea laurifolia*	
		Coccodiniaceae	*Microxiphium*	*Olea europaea* sbsp *africana*	Quinolactacin A2, Radicidin
		Incertae sedis	*Unidentified Chaetothryriales*	*Elegia capensis*	
	Eurotiales	*Aspergillaceae*	*Penicillium* (n=4)	*Protea laurifolia*	Antibiotic NG 011, Secalonic acid C, Chaetoglobusin P/N and K/L
	Phaeomoniellales	*Phaeomoniellaceae*	*Phaeomoniella* (n=2)	*Protea laurifolia*	
			Unidentified Phaeomoniellales	*Protea laurifolia*	Cerebroside C, Coriolide, Lecanorin
		Celotheliaceae	*Neophaeomoniella*	*Diosma subulata*	
Pezizomycetes	*Pezizales*	*Sarcosomataceae*	*Conoplea*	*Protea repens*	

Pezizomycetes	*Pezizales*	*Sarcosomataceae*	*Plectania* (n=2)	*Brabejum stellatifolium* and *Protea repens*	Isogaliellalactone
Cystobasidiomycetes	*Cystobasidiales*	*Cystobasidiaceae*	*Cystobasidium*	*Elegia capensis*	Citrinin
Umbelopsidomycetes	*Umbelopsidales*	*Umbelopsidaceae*	*Umbelopsis*	*Olea europaea sbsp africana*	
Wallemiomycetes	*Wallemiales*	*Wallemiaceae*	*Wallemia*	*Protea repens*	
Microbotryomycetes	*Sporidiobolales*	*Sporidiobolaceae*	*Sporobolomyces*	*Protea laurifolia*	
Orbiliomycetes	*Orbiliales*	*Orbiliaceae*	*Orbilia*	*Protea repens*	
Incertae sedis	*Incertae sedis*	*Incertae sedis*	*Anthopsis* (n=2)	*Chrysanthemoides monilifera*	
			Ochroconis	*Protea repens*	
			Polyscytalum (n=2)	Oak tree *Quercus* sp.	Mellein, 4/3Hydroxymellein
			Pseudosigmoidea	Oak tree *Quercus* sp.	
			Rhexodenticula	*Protea laurifolia*	
			Unidentified (n=10)	Fynbos of restios, *Chrysanthemoides monilifera*, *Elegia capensis*, *Protea repens*, *Olea europaea sbsp africana*	Connatusin B, N-deoxyakanthomycin, 11-Hydroxycurvularin, MDN-0101 [42], Nemanolone A, Pandangolide 2, Preussomerin L, Questiomycin A, Resorcylide
